# Survival and clinical outcomes of patients with melanoma brain metastasis in the era of checkpoint inhibitors and targeted therapies

**DOI:** 10.1186/s12885-018-4374-x

**Published:** 2018-04-27

**Authors:** Elham Vosoughi, Jee Min Lee, James R. Miller, Mehdi Nosrati, David R. Minor, Roy Abendroth, John W. Lee, Brian T. Andrews, Lewis Z. Leng, Max Wu, Stanley P. Leong, Mohammed Kashani-Sabet, Kevin B. Kim

**Affiliations:** Center for Melanoma Research and Treatment, California Pacific Medical Center Research Institute, 2100 Webster Street, Suite 326, San Francisco, CA 94115 USA

**Keywords:** Melanoma, Brain, Metastasis, Checkpoint inhibitors, BRAF

## Abstract

**Background:**

Melanoma brain metastasis is associated with an extremely poor prognosis, with a median overall survival of 4–5 months. Since 2011, the overall survival of patients with stage IV melanoma has been significantly improved with the advent of new targeted therapies and checkpoint inhibitors. We analyze the survival outcomes of patients diagnosed with brain metastasis after the introduction of these novel drugs.

**Methods:**

We performed a retrospective analysis of our melanoma center database and identified 79 patients with brain metastasis between 2011 and 2015.

**Results:**

The median time from primary melanoma diagnosis to brain metastasis was 3.2 years. The median overall survival duration from the time of initial brain metastasis was 12.8 months. Following a diagnosis of brain metastasis, 39 (49.4%), 28 (35.4%), and 24 (30.4%) patients were treated with anti-CTLA-4 antibody, anti-PD-1 antibody, or BRAF inhibitors (with or without a MEK inhibitor), with a median overall survival of 19.2 months, 37.9 months and 12.7 months, respectively. Factors associated with significantly reduced overall survival included male sex, cerebellar metastasis, higher number of brain lesions, and treatment with whole-brain radiation therapy. Factors associated with significantly longer overall survival included treatment with craniotomy, stereotactic radiosurgery, or with anti-PD-1 antibody after initial diagnosis of brain metastasis.

**Conclusions:**

These results show a significant improvement in the overall survival of patients with melanoma brain metastasis in the era of novel therapies. In addition, they suggest the activity of anti-PD-1 therapy specifically in the setting of brain metastasis.

## Background

Brain metastases are common in patients with advanced melanoma and are a frequent cause of death in patients with this disease [1]. Nearly 20% of patients are found to have brain metastasis at the time of diagnosis of metastatic melanoma, and more than 50% develop brain metastasis during the course of the disease [2–5]. Brain metastasis is associated with a poor prognosis, with median overall survival from diagnosis of brain metastasis in the range of 17–22 weeks [2, 4, 6, 7]. Until recently, the management of melanoma brain metastasis has included surgical resection, stereotactic radiosurgery, whole-brain radiation therapy, and/or cytotoxic chemotherapy [8, 9], without a clear change in the natural history of melanoma brain metastasis.

Since 2011, a number of targeted therapies, including BRAF inhibitors and MEK inhibitors, and checkpoint inhibitors, such as anti-CTLA4 antibody and anti-PD-1 antibodies, have been approved by the Food and Drug Administration (FDA) in the United States because of their significant survival benefit, and have emerged as new standard therapies. As of 2016, the median survival duration of patients with unresectable or metastatic melanoma approaches nearly 2 years with these novel drug therapies [10–12], compared to 6–9 months with traditional cytotoxic chemotherapy [13–15]. However, the impact of these new drugs on the clinical outcome and survival of patients with brain metastasis is not well known, although a number of prospective clinical trials have shown promising clinical activity of these agents in the setting of brain metastasis [1, 3]. However, these studies do not address the survival outcomes of patients who are not candidates for systemic therapies or clinical trials. Therefore, there is a lack of current survival data in patients with melanoma brain metastasis in a real world situation in the modern era.

Here, we report the findings of our retrospective analysis of outcomes of patients diagnosed with melanoma brain metastasis in the era of the novel targeted therapies and immunotherapies.

## Methods

We searched the Institutional Tumor Registry Database and Melanoma Database at California Pacific Medical Center and San Francisco Oncology Associates for patients with diagnosis of metastatic melanoma to the brain. Under an institutional review board-approved protocol, we performed a retrospective medical record review of all melanoma patients with brain metastases. Because checkpoint inhibitors and BRAF inhibitors have been approved by the FDA beginning in 2011, we limited our search to those who were diagnosed with brain metastasis between January of 2011 and June of 2015. Patients were eligible for inclusion in the study if they had at least 6 months of adequate follow up evaluation since the time of initial brain metastasis, unless they had died within 6 months after the initial date of initial brain metastasis. The final analysis was performed in December of 2016. We utilized Cox regression for univariate and multivariate analyses of the potential association between various clinical or histological factors with overall survival. Kaplan-Meier analysis was used to determine the overall survival of patients, including differences between specific subgroups of patients.

## Results

### Clinical characteristics of the patients with brain metastasis

A total of 79 patients were identified for this analysis. The demographic and baseline characteristics of the patients are described in Table 1. The median time from primary melanoma diagnosis to brain metastasis was 3.2 years (range, 0–29.8 years), and the median time from stage IV diagnosis to brain metastasis was 2 months (range, 0–103 months). Forty (50.6%) patients had prior extracranial metastasis at the time of initial brain metastasis; 28 (35.4%) had concurrent extracranial metastasis at the time of brain metastasis; and 5 (6.3%) patients developed extracranial metastasis subsequently, defined as at least 1 month after initial diagnosis of brain metastasis. Six (7.6%) patients had brain metastasis as the only site of distant metastasis until death or at the time of the analysis.

**Table 1:** Patient characteristics and treatment (*n* = 79)

Characteristic at the time of initial brain metastasis	No. of patients (%)
Median age (range), years	63 (17–91)
Sex	
Male	53 (67.1%)
Stage prior to initial brain metastasis,	
I/II	13 (16.4%)
III	13 (16.4%)
IV (M1a)	6 (7.6%)
IV (M1b)	8 (10.1%)
IV (M1c)	25 (31.6%)
Unknown primary melanoma/Data not available	14 (17.7%)
Intracranial site of metastasis^a^	
Cerebrum	72 (91.1%)
Cerebellum	17 (21.5%)
Pons	7 (8.9%)
Leptomeninges	2 (2.5%)
Unknown	1 (1.3%)
Number of brain metastasis^a^	
1	39 (49.4%)
2–3	15 (19%)
4–5	6 (7.6%)
6–9	3 (3.8%)
≥ 10	12 (15.2%)
Unknown	4 (5.0%)
Size of the largest brain metastasis^a^	
≤ 10 mm	25 (31.7%)
> 10–30 mm	32 (40.5%)
> 30–50 mm	15 (19.0%)
> 50 mm	3 (3.8%)
Unknown	4 (5.0%)
Symptomatic from brain metastasis	
Yes	36 (45.6%)
Sites of extracranial metastatic organs^a^	
Lung	22 (27.9%)
LN/Soft tissue	21 (26.6%)
Skin/SQ	15 (19.0%)
Bone	12 (15.2%)
Liver	8 (10.1%)
Adrenal gland	3 (3.8%)
V600 BRAF mutation	
Mutated	29 (36.7%)
Wild type	38 (48.1%)
Unknown	12(15.2%)
Number of systemic therapy given after a diagnosis of brain metastasis	
1	28 (35.4%)
2	22 (27.8%)
3+	20 (25.3%)
Info not available	9 (11.4%)
Type of systemic therapy given after a diagnosis of brain metastasis	
Ipilimumab	39 (49.4%)
Anti PD-1 antibody	28 (35.4%)
Concurrent Nivolumab/Ipilimumab	8 (10.1%)
BRAF inhibitor (+/− MEK inhibitor) only	24 (30.4%)
Cytotoxic chemotherapy	35 (44.3%)
Interleukin-2-based biochemotherapy	10 (12.7%)
Sequence of novel drug therapy after a diagnosis of brain metastasis^b^	
Checkpoint inhibitor followed by BRAF inhibitor (+/− MEK inhibitor)	11 (13.9%)^b^
BRAF inhibitor (+/− MEK inhibitor) followed by checkpoint inhibitor	3 (3.8%)^b^

^a^at the time of initial brain metastasis diagnosis

^b^A total of 14 patients were treated with both checkpoint inhibitor and BRAF inhibitor (+/− MEK inhibitor) after a diagnosis of brain metastasis

The cerebrum was the most common site of brain metastasis (72 patients [91.1%]), and 21.5% and 8.9% patients had metastasis to the cerebellum and pons, respectively. Thirty-nine (49.4%) had a solitary brain metastasis at the initial brain metastasis diagnosis, and the largest size of the initial brain metastasis was 10 mm or less in 31.7%. Thirty-six patients (45.6%) had neurological symptoms associated with brain metastasis. Forty-nine (62.0%) of the 79 patients had received systemic therapy prior to or at the time of brain metastasis, including checkpoint inhibitors, targeted drugs, cytotoxic chemotherapy and/or cytokine therapy.

### Treatment modalities

Thirty-four patients (43.0%) underwent craniotomy for the management of brain metastasis, and 54 (68.4%) were treated with stereotactic radiosurgery. After diagnosis of brain metastasis, 39 (49.4%), 28 (35.4%), and 24 (30.4%) patients were treated with anti-CTLA-4 antibody, anti-PD-1 antibody, or BRAF inhibitors (with or without a MEK inhibitor), respectively. Thirty-five (44.3%) and ten (12.7%) patients were treated with cytotoxic chemotherapy and interleukin-2 treatment, respectively.

### Survival and clinical outcome

**Fig. 1:**
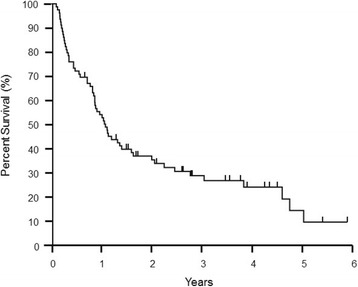
Kaplan-Meier curve of overall survival melanoma brain metastasis cohort (*n* = 79)

**Fig. 2:**
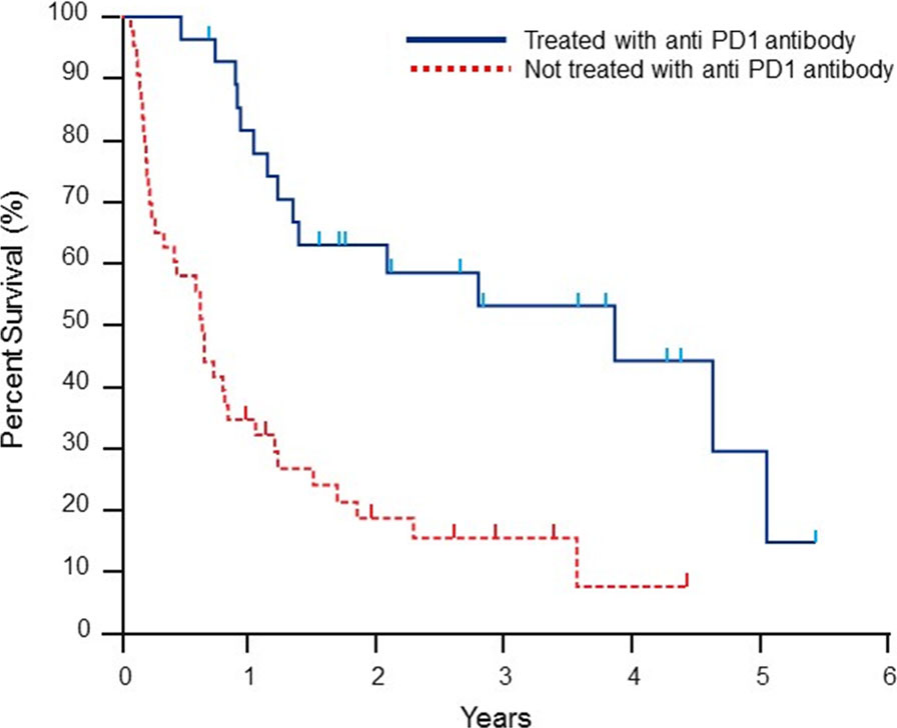
Kaplan-Meier curves of overall survival by presence or absence of anti-PD-1 antibody therapy

Fifty-nine (74.7%) patients had died of melanoma progression at the time of the analysis, among which 32 (40.5%) died with progressing brain metastases. The median overall survival duration from the time of initial brain metastasis was 12.8 months (range, 1.1–71.9 months) (Fig. 1), and the median overall survival duration from the time of initial melanoma diagnosis was 60.5 months (5.5–367.1 months) for all 79 patients. The median overall survival durations from the time of craniotomy and stereotactic radiosurgery were 17.3 months (2.4–60.7 months) and 15.4 months (1.2–71.8 months), respectively. The median survival durations of patients who received anti-CTLA-4 antibody, anti-PD-1 antibody and BRAF inhibitor (with or without MEK inhibitor) after the diagnosis of brain metastasis were 19.2 months (1.2–65.0 months), 37.9 months (5.3–65.0 months) and 12.7 months (2.7–70.9 months), respectively. Tables 2 and 3 describe the outcomes of the entire cohort as well as specific subsets of patients. Figures 1 and 2 illustrate the Kaplan-Meier curves of overall survival for all patients and for those who were treated with or without anti-PD-1 therapy, respectively.

**Table 2:** Overall survival outcome data

From the time of	Median OS (range), months
initial brain metastasis (*n* = 79)	12.8 (1.1–71.9)
initial melanoma diagnosis (*n* = 79)	60.5 (5.5–367.1)
the first craniotomy (*n* = 34)	17.3 (2.4–60.7)
the first stereotactic radiosurgery (*n* = 54)	15.4 (1.2–71.8)
the first whole brain radiation therapy (*n* = 16)	6.8 (2.2–12.5)

OS, overall survival

**Table 3:** Subset analyses of overall survival outcomes

OS from the time of initial brain metastasis in patients who:	Median OS, (range), months
Were treated with anti-CTLA-4 antibody therapy	
Before the initial brain metastasis (*n* = 29)	10.5 (2.0–55.3)
After the initial brain metastasis (*n* = 39)	19.2 (1.2–65.0)
Were treated with anti-PD-1 antibody therapy	
Before the initial brain metastasis (*n* = 1)	8.5
After the initial brain metastasis (*n* = 28)	37.9 (5.3–65.0)
Were treated with BRAF and/or MEK inhibitor therapy	
Before the initial brain metastasis (*n* = 16)	10.9 (2.1–55.3)
After the initial brain metastasis (*n* = 24)	12.7 (2.7–70.9)

### Predictive factors for overall survival

We analyzed the potential association between several factors and survival using univariate Cox regression of overall survival (Table 4). Intriguingly, of factors in the primary tumor, increased levels of tumor-infiltrating lymphocytes showed a trend toward improved survival in patients with brain metastasis. Several clinical factors were found to be significantly associated with overall survival in patients with brain metastasis by univariate analysis (Table 4). Factors associated with shorter overall survival included male sex, cerebellar involvement, higher number of metastatic brain tumors, concurrent presence of adrenal metastasis, or treatment with whole-brain radiation therapy. Factors associated with longer overall survival were treatment with craniotomy, stereotactic radiosurgery, or anti-PD-1 antibody therapy after initial diagnosis of brain metastasis.

**Table 4:** Univariate Cox regression analysis of association of various clinical factors with overall survival in melanoma patients with brain metastasis

Variable	Chi-squared	Hazard ratio	*P* value
Gender (male)	4.03	1.85	0.045*
Age	0.826	1.01	0.36
TIL level	2.23	0.628	0.135
Number of positive regional lymph nodes	3.13	1.05	0.077
Characteristics of brain metastasis			
Cerebellar metastases	6.43	2.19	0.011*
Midbrain/pons metastasis	3.24	2.09	0.072
Leptomeningeal disease	0.04	1.07	0.846
Other site metastasis	0.03	0.92	0.867
Size of largest brain metastases	0.816	1.01	0.367
Number of brain metastases	18.8	1.19	< 0.00005*
Presence of neurological symptoms	1.55	2.51	0.214
Required steroid for brain metastasis	1.55	2.51	0.214
Extracranial Metastasis			
Presence of extracranial metastasis	2.29	1.49	0.13
Presence of liver metastasis	2.25	1.94	0.134
Presence of lung metastasis	0.527	1.30	0.468
Presence of bone metastasis	0.06	1.09	0.813
Presence of adrenal metastasis	5.39	4.50	0.02*
Therapy			
Craniotomy	10.0	0.418	0.0015*
Stereotactic radiosurgery	7.72	0.434	0.0055*
Whole brain radiation therapy	16.2	3.85	0.0001*
Number of prior systemic therapies	0.613	1.09	0.434
Anti-CTLA4 antibody after BM	0.658	0.79	0.417
Anti-PD-1 antibody after BM	10.0	0.376	0.0016*
BRAF inhibitor after BM	0.05	1.07	0.82

BM, initial diagnosis of brain metastasis;

*p-value is < 0.05

Multivariate analysis of all eight factors revealed cerebellar involvement, craniotomy, and adrenal involvement as independently predictive of survival (Table 5). There was trend toward significance for treatment with anti-PD-1 antibody (*P = 0.055*).

**Table 5:** Multivariate Cox regression analysis of association of various clinical factors with overall survival in melanoma patients with brain metastasis

Variable	Chi-squared	Hazard ratio	*P* value
Gender	0.89	1.93	0.35
Presence of cerebellar metastasis	6.74	4.61	0.009*
Number of brain metastases	0.60	0.93	0.44
Presence of adrenal Metastasis	4.89	9.94	0.027*
Craniotomy	4.45	0.27	0.035*
Stereotactic radiosurgery	0.92	0.51	0.34
Treated with whole brain radiation therapy	1.08	2.13	0.298
Treatment with anti-PD-1 antibody	3.68	0.34	0.055

*p-value is < 0.05

## Discussion

Patients with metastatic melanoma to the brain have been considered to have an extremely poor prognosis with a short median overall survival, and in a vast majority of cases, deaths observed are due to disease progression in the brain. Sampson et al. showed that brain metastasis was responsible for death in 94.5% of patients in their retrospective analysis of patients with melanoma brain metastasis [16]. Although our database do not have the detailed information regarding neurological symptoms at the time of death, we believe that most of our patients have died of the brain metastasis, similar to the historical data. In our study, we report a longer survival of patients with brain metastasis in the era of novel checkpoint inhibitors and BRAF inhibitor-based targeted therapies. The median overall survival duration among all 79 analyzed patients was longer than 1 year. Specifically, among those who were treated with anti-PD-1 antibody, the median survival was nearly 3 years.

Most of the available literature regarding the survival of melanoma patients with brain metastasis was published prior to 2011, when BRAF inhibitor-based therapies and checkpoint inhibitors became available as standard therapies for advanced melanoma. Prior to 2011, most patients were treated with stereotactic radiosurgery to metastatic lesions, whole brain radiation, and/or cytotoxic chemotherapy with or without cytokines, such as interferon-alpha and interleukin-2. As our data show, a vast majority of patients who were diagnosed with brain metastasis since 2011 were treated with the BRAF (and/or MEK)-targeting kinase inhibitors and/or checkpoint inhibitors. Therefore, the prolonged overall survival of the patients in our analysis is most likely due to the clinical benefit of these novel targeted and/or immunotherapeutic drugs. Specifically, patients who were also treated with anti-PD-1 antibody therapy had significantly longer survival, compared to those who had not. Since most patients diagnosed with brain metastasis on or after 2013, when anti-PD-1 antibodies were approved by the FDA, received either nivolumab or pembrolizumab, the longer survival duration is most likely due to the anticancer activity of anti-PD-1 antibody rather than biased patient selection for the treatment, even in the setting of brain metastasis.

One might wonder whether the change in the pattern of therapeutic modality other than the checkpoint inhibitors and BRAF inhibitor-based targeted therapies contributed the longer survival of the patients in the recent years. Davies and colleagues showed that there was no significant difference in the median overall survival duration between melanoma patients with brain metastasis diagnosed prior to 1996 and those diagnosed between 1996 and 2004 [4]. Although a stereotactic radiosurgery was likely used more frequently in the latter time period, there was no significant survival improvement in the overall patients. In their study, the median overall survival duration in patients who were initially treated with a stereotactic radiosurgery was 7.69 months whereas the median survival was 15.4 months post stereotactic radiosurgery in our study. Therefore, we believe that evolution in the pattern of therapeutic modality for brain metastasis has a minimal impact in overall survival in this patient population until the availability of the checkpoint inhibitors and the targeted therapy drugs.

Our findings showed an improved outcome in this patient population compared to a recent meta-analysis performed by Spagnolo et al., which analyzed 22 clinical studies (including 8 phase I-II studies and 14 “real world” expanded-access program studies), which included 2153 melanoma patients with brain metastasis in the era of MAP-kinase inhibitors and checkpoint inhibitors [2]. In their analysis, the median overall survival of all analyzed patients was 7.9 months and 7.7 months for the phase I-II studies and “real-world” studies, respectively. Although the authors had an intention to report the survival in a “real-world” situation, their results do not necessarily represent all patients with brain metastasis because their findings are based on patients who were able to enroll in the clinical studies, whether or not they are expanded access program studies. It is most likely that only a subset of patients with brain metastasis would meet the eligibility criteria for each study. In addition, the overall survival duration in their analysis was measured from the time of initiation of the novel systemic therapy, not from the time of initial brain metastasis; therefore, patients with brain metastasis who were treated only with local therapy, such as craniotomy, stereotactic radiosurgery or whole brain radiation, without the novel systemic therapy, were not included in their analysis. Lastly, most patients were treated with the targeted therapies and/or ipilimumab, and only 18 of 2153 patients in their analysis received anti-PD-1 antibody therapy. Since anti-PD-1 antibody therapies have shown to be superior to ipilimumab [17, 18], their findings are not likely to represent the true clinical outcome in the current era, where anti-PD-1 antibody therapy has replaced ipilimumab alone as the first-line standard therapy in patients with advanced melanoma.

Our results suggest that the targeted and checkpoint inhibitor drugs have meaningful clinical benefit in patients with brain metastases. This phenomenon was initially observed in phase II studies of ipilimumab and BRAF inhibitors, in which significant regression of active metastatic brain lesions occurred following with treatment with these drugs [1, 3]. More recently, a number of prospective phase II clinical studies have demonstrated that these novel drugs have significant clinical activity in melanoma patients with active brain metastasis [19–21]. The response rates of a combination of nivolumab and ipilimumab were 42%–55% in two of the studies [19, 20] and a combination of dabrafenib and trametininb had an objective response rate of 58% [21]. Unfortunately, a clinical response to a specific therapy could not be appropriately evaluated in our study because most patients were treated with a multimodality therapy, such as a local brain therapy (craniotomy, stereotactic radiosurgery and/or whole brain radiation) administered either concurrently with or shortly followed by a systemic therapy. We believe that our pattern of treatment in patients with active brain metastasis is typical of most community oncology practices, including patients treated outside of a clinical trial.

Our results suggest that the overall survival is especially poor in patients with cerebellar metastasis or in those with concurrent adrenal metastasis. The shorter survival duration for patients with cerebellar involvement in our analysis is consistent with previously reported data [22–24]. Our finding of poor prognosis in those with concurrent brain and adrenal metastasis is interesting and has not been previously been reported. However, due to the small number of patients with this finding, it deserves confirmation in a separate cohort of patients. Similarly, our findings of no significant prognostic impact of the presence of leptomeningeal involvement may be due to the inclusion of a small number of such patients in our analysis.

Our study is the first to show the impact of novel targeted drugs and immunotherapies on the overall survival of patients with brain metastasis. Specifically, our study is the first to show a significantly improved survival of patients receiving anti-PD-1 therapy following the development of brain metastasis. It is particularly interesting that the median survival duration was nearly 3 years in those patients who were treated with anti-PD-1 antibody therapy. Although our study included a relatively small number of patients at a tertiary referral center, our results are very encouraging and show an altered natural history of melanoma brain metastasis, which deserves confirmation in additional, larger cohorts of melanoma patients.

## Conclusions

We show significantly improved survival of melanoma patients with brain metastasis in the era of novel targeted and immunotherapeutic drugs. The median overall survival of those with melanoma brain metastasis is longer than 1 year, and nearly 3 years for those who were treated with anti-PD-1 antibody. These results strongly suggest the impact of novel immunotherapies on prolonging the survival of these patients.
